# Notes from Hospital Practice

**Published:** 1878-01

**Authors:** 


### NOTES FROM HOSPITAL PRACTICE.

*Carcinoma of Tongue.—Partial Ablation.*

A remarkable case of epithelioma of the tongue recently came under the notice of the staff of the Albany (N. Y.) City Hospital. The patient, J. D., æt. 74, Irish by birth, and mentally infirm, came to the hospital in January, 1877. He gave no history of his lesion, but a malignant growth was discovered on the under surface of the left side of the tongue, involving nearly one-half of the organ. Ulceration had already begun, and had resulted in a considerable excavation, while the body of the tongue was hard and semi-indurated. There was the characteristic pain of carcinoma, sharp and lancinating at night after exertion, heavy and dull in the early part of the day. The tongue still retained its normal size, and preserved its natural color, save in the diseased portion, where it was superficially of a brownish-gray hue. Deglutition was impaired even then, and articulation was difficult, every word being muffled and indistinct. The general health, considering the age of the patient, was far from good, and though he was in good spirits, it was clear that the disease was progressing.

Dr. Vanderveer, of the staff, diagnosticated a carcinomatous lesion, and it may be said that any other diagnosis would have been impossible. It was a case of carcinoma, of epithelial type, in a most difficult location for topical treatment, already so far advanced as to present formidable features.

The treatment proposed was necessarily severe. A cure was virtually impossible. Alleviation was the only alternative.

Electrolytic treatment by the use of the galvanic cautery was tried on six several occasions. At first the advance of the disease was arrested, and seemed under temporary control; but the disease spread insidiously, gradually involving more and more of the tissues, and provoking sympathetic enlargement of the cervical glands. On Oct. 27, the cautery was used for the seventh time, and then abandoned as useless. In the ten months that had passed since the patient's entrance to the hospital, although the carcinomatous cachexia had become more actively developed, his systemic condition was better than one year before.

A consultation of the hospital staff was held, and the operation of ablation of the tongue was deemed advisable so far as to remove the diseased growth. This operation was performed Nov. 14, by Dr. Albert Vanderveer, assisted by Drs. Ward and Hailes.

The patient anæthetised, the tissues from the middle of the lower lip were divided in a perpendicular line to the symphysis mentis, and reflected. The inferior maxilla was then sawn in two at the symphysis, after the method of Syme. The forefinger of the operator was then inserted into the opening under the tongue, and the knife thus guided, used to divide the mucous membrane of the mouth and the sub-lingual muscles.

The lingual artery of the affected side was ligated and divided, the hemorrhage being thus almost entirely arrested. These preparatory steps being successfully performed, the diseased growth was laid before the surgeon ready for action.

The galvanic cautery, in the form of a loop of platinum wire, was applied around the diseased part of the organ, and by two operations the morbid tissues effectually cut away, about one-third of the tongue being left. The hemorrhage was inconsiderable, and the operation satisfactory in all respects to the surgeon and his assistants. A number of physicians were present to witness the operation.

The patient presents a favorable prognosis, and permanent success may be hoped for. W. H. M.

ALBANY, N. Y., Nov. 15.

